# Assimilation of phthalate esters in bacteria

**DOI:** 10.1007/s00253-024-13105-6

**Published:** 2024-03-27

**Authors:** Pei Qiao, Tongtong Ying, Mengjie Gu, Jiahong Zhu, Chengyu Mei, Tong Hu, Tengfei Liu, Haixia Wang, Weihong Zhong

**Affiliations:** College of Biotechnology and Bioengineering, Zhejiang University of Technology, Hangzhou, 310014 China

**Keywords:** Phthalate esters, Transmembrane assimilation, Spontaneous permeation, Membrane facilitated outer/inner membrane transport

## Abstract

**Abstract:**

The massive usage of phthalate esters (PAEs) has caused serious pollution. Bacterial degradation is a potential strategy to remove PAE contamination. So far, an increasing number of PAE-degrading strains have been isolated, and the catabolism of PAEs has been extensively studied and reviewed. However, the investigation into the bacterial PAE uptake process has received limited attention and remains preliminary. PAEs can interact spontaneously with compounds like peptidoglycan, lipopolysaccharides, and lipids on the bacterial cell envelope to migrate inside. However, this process compromises the structural integrity of the cells and causes disruptions. Thus, membrane protein-facilitated transport seems to be the main assimilation strategy in bacteria. So far, only an ATP-binding-cassette transporter PatDABC was proven to transport PAEs across the cytomembrane in a Gram-positive bacterium *Rhodococcus jostii* RHA1. Other cytomembrane proteins like major facilitator superfamily (MFS) proteins and outer membrane proteins in cell walls like FadL family channels, TonB-dependent transporters, and OmpW family proteins were only reported to facilitate the transport of PAEs analogs such as monoaromatic and polyaromatic hydrocarbons. The functions of these proteins in the intracellular transport of PAEs in bacteria await characterization and it is a promising avenue for future research on enhancing bacterial degradation of PAEs.

**Key points:**

• *Membrane proteins on the bacterial cell envelope may be PAE transporters.*

• *Most potential transporters need experimental validation.*

## Introduction

Plastics have been widely produced and used to facilitate people’s daily lives since the 1950s (Sardon and Dove 2018). Accordingly, phthalate esters (PAEs) are being massively used as plasticizers to enhance the flexibility and malleability of plastics (Liu et al. 2021). PAEs are also used as additives in the manufacturing of pesticides, paints, and cosmetic products (Ramzi et al. 2018). With global plastic production exceeding 150 million tons annually and PAE consumption reaching 6–8 million tons per year, PAEs are projected to account for 50–55% of global plasticizer usage despite usage restrictions (Tran et al. 2022; Zhang et al. 2020a).

PAEs form weak non-covalent bonds with polymeric matrices, allowing for easy leaching and environmental migration. Consequently, they accumulate in ecosystems, posing long-term exposure risks to organisms and humans (Giuliani et al. 2020). PAEs are known as endocrine-disrupting substances (EDCs) and potential carcinogens, with chronic exposure linked to reproductive toxicity, hormonal disturbances, hepatocellular tumors, renal organ damage, increased breast cancer risk, and impaired fetal neurodevelopment (Balabanič et al. 2021; Du et al. 2020; Gao et al. 2018; Li et al. 2021; Tian et al. 2022). To address the growing concern of PAEs pollution, United States Environmental Protection Agency (USEPA), European Union (EU), and Chinese National Environmental Monitoring Center (CNEMC) have classified dimethyl phthalate (DMP), diethyl phthalate (DEP), dibutyl phthalate (DBP), butyl benzyl phthalate (BBP), di(2-ethylhexyl) phthalate (DEHP), and di-n-octyl phthalate (DnOP) as priority pollutants and restricted their usage (Hu et al. 2021).

Apart from usage control, the removal of PAEs from the environment is being actively pursued. Biodegradation is regarded as a primary approach due to its efficiency, environmental friendliness, and cost-effectiveness (Chen et al. 2021). Since its emergence in the 1980s, biodegradation of PAEs has received great attention, and extensive research has been conducted to understand and harness this process. PAE-degrading bacteria have been isolated from ecosystems like soil, water, sediments, and animal gastrointestinal tracts (Chen et al. 2021). Over 80 PAE-degrading bacterial strains belonging to 36 genera, predominantly *Pseudomonas*, *Gordonia*, *Rhodococcus*, *Sphingomonas*, and *Baicillus*, have been identified (Feng et al. 2021; Hu et al. 2022; Huang et al. 2019; Liu et al. 2023; Mahajan et al. 2019; Zhao et al. 2018).

The metabolism of various PAEs has been exhaustively investigated. Including some of our research, these works are mainly focused on the enzymatic hydrolysis of PAEs and the mineralization of phthalate (PA) (Hu et al. 2022; Liu et al. 2020; Ren et al. 2021). However, research on the permeation process and assimilation mechanisms of PAEs into bacterial cells is relatively weak (Bao et al. 2022; Luo et al. 2017; Qiu et al. 2020). This knowledge deficiency represents a major limitation in the study of PAE biodegradation. Hence, this review aims to offer a comprehensive overview of the current advancements made in the bacterial assimilation process of PAEs and also provides insights into the future directions and challenges for further research in this field.

## Cell wall transport of PAEs in bacteria

**Fig. 1:**
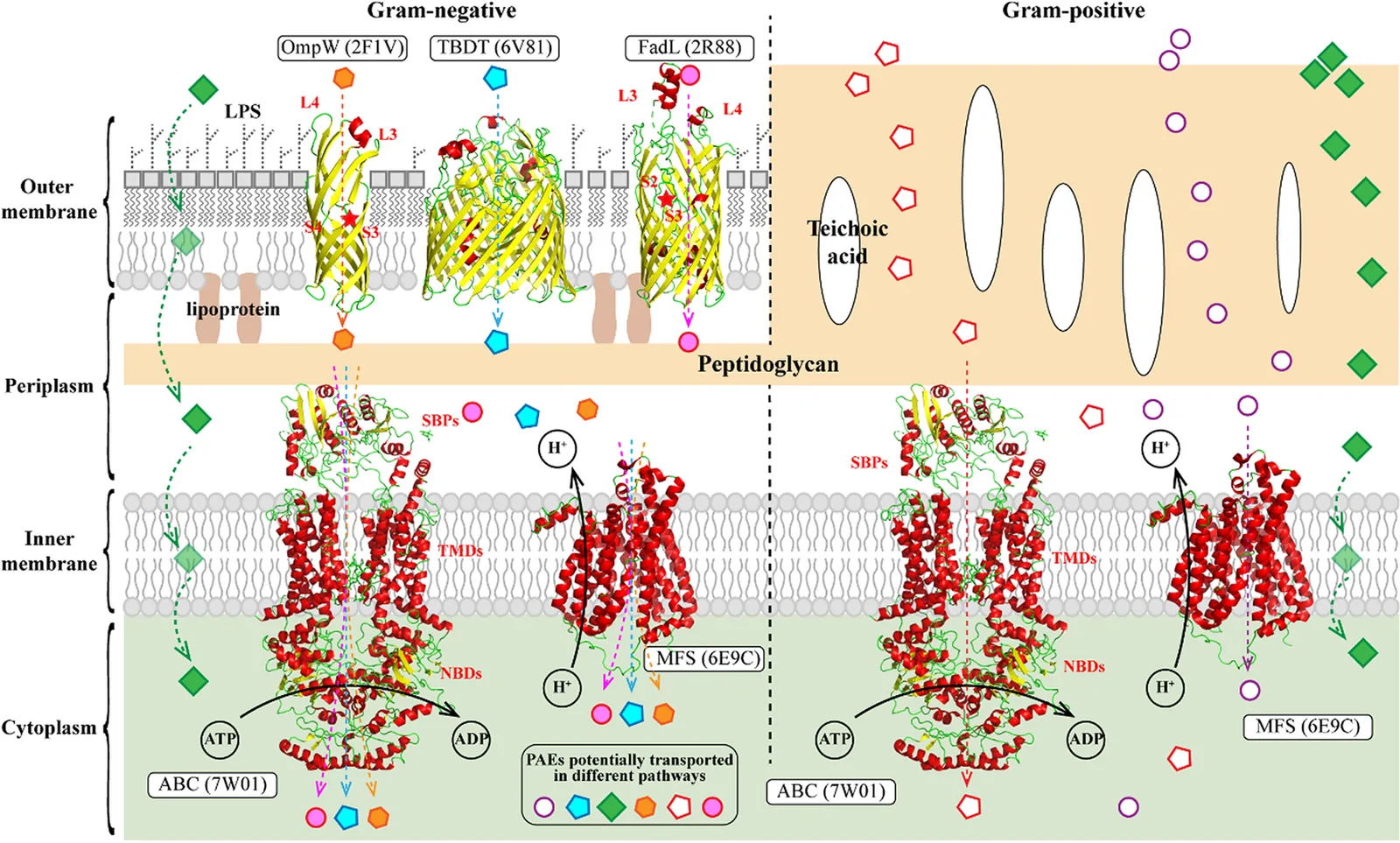
Schematic representation of PAE transmembrane assimilation in both Gram-negative and -positive bacteria with or without the help of membrane proteins. The space in light yellow color represents the peptidoglycan structure on the bacterial cell wall. The white oval with a black edge represents teichoic acid. The space in the light green color represents the cytoplasm. The cyan pentagon, green square, brown hexagon, pink circle, white pentagon with red edge, and white circle with purple edge represent PAEs that are potentially transported in different pathways. ABC represents ABC transporter (PDB: 7W01; SBPs mean substrate binding proteins; TMDs mean transmembrane domains; NBDs mean nucleotide-binding domains. MFS represents the major facilitator superfamily (PDB: 6E9C). OmpW represents outer membrane protein W (PDB: 2F1V). TBDT represents a TonB-dependent transporter (PDB: 6V81). FadL represents the outer membrane protein for long-chain fatty acids (PDB: 2R88)

The cell wall is the first barrier against PAE bacterial uptake. Its structure is relatively simple in Gram-positive bacteria, mostly constituted by peptidoglycan (60 to 95%) and teichoic acid (10% to 30%). The cell wall in Gram-negative bacteria is relatively complicated, with a thinner peptidoglycan layer covered by an outer membrane (Planas 2022; Scheffers and Pinho 2005). The outer membrane contains lipopolysaccharides (LPS), phospholipids, outer membrane proteins, porins, and lipoproteins (Fig. 1). Existing evidence suggests that the PAE transport through the cell wall seems to be a comprehensive process that involves direct permeation and membrane protein-facilitated uptake.

### PAEs interact with peptidoglycan, proteins, and lipids on bacterial cell walls through hydrophobicity

A study on the adsorption of DBP (1–15 mg/L) by Gram-negative bacteria *Burkholeria cepacian* using Fourier transform infrared (FTIR) spectroscopy showed that DBP adsorption by *B. cepacian* was mainly due to the hydrophobic interaction between PAEs and the cell envelope of Gram-negative bacteria that contains outer membrane lipids, proteins, and peptidoglycan underneath. The DBP adsorption capacity of *B. cepacian* slightly increased when the hydrated and loosely arranged cell capsule outside the outer membrane was removed. Similarly, the DBP adsorption capacity increased when peptidoglycan was exposed with the removal of the outer membrane. (Luo et al. 2017). In another study on DBP (1–15 mg/L) adsorption by *B. cepacia*, the researchers found that the hydrophobic interaction between DBP and the cell wall played a crucial role. They also identified that outer membrane proteins and phosphate lipids are the key participants in binding DBP to the cell’s outer membrane (Lu et al. 2016). A Gram-positive bacterium *Lactobacillus acidophilus* was reported to bind and remove DBP. DBP was bound to the peptidoglycan on the bacterial cell surface and hydrophobic interaction was the main binding force (Zhao et al. 2021). The uptake of DBP (500 mg/L) by *Novosphingobium* sp. DNB-S3 (Gram negative) was investigated in a more recent study. The authors compared the morphological change on the outer membrane of DNB-S3 using infrared spectroscopy and Rama spectroscopy and observed an externally exposed outer membrane from cellular structure, inducing an increased cell surface hydrophobicity that promoted DBP uptake efficiency (Feng et al. 2020). By applying anthraquinone-2,6-disulfonate (AQDS) to a DBP-degrading bacteria *Enterobacter* sp. DNB-S2 (Gram negative), Zhang et al. successfully enhanced the biodegradation capacity of the strain for DBP (500 mg/L). Specifically, AQOS increased the hydrophobic level and the ratio of unsaturated fatty acids of the cell surface. These cell surface modifications enhanced the chemotactic ability of strain DNB-S2 to the hydrophobic molecule DBP and the fluidity of the cell outer membrane (Zhang et al. 2020b). Besides, *Stenotrophomonas maltophilia* possess hydrophobic glycosphingolipids (GSLs) on their outer membrane, instead of lipopolysaccharides (LPS) found in typical Gram-negative bacteria (Li et al. 2020). These GSLs could facilitate the interaction and absorption of hydrophobic compounds, such as PAEs to enhance their degradation capabilities (Li et al. 2020).

### PAEs adsorption damages bacterial cell wall

Direct evidence on the changed morphology of cell wall and cell surface hydrophobicity upon PAE adsorption was observed by Wang et al. on the degradation of DMP by *Pseudomonas fluorescens* (Gram negative). The scanning electron microscopy (SEM) observation on *P. fluorescens* showed that DMP (20–40 mg/L) treatment introduced morphological abnormalities and damaged cell walls and cell membranes. Although *P. fluorescens* could grow with DMP as the source carbon, DMP exposure damaged the cell surface and caused nucleotide leakage, restricting the growth of *P. fluorescens* in turn*.* In addition to the changed cell surface permeability, DMP has induced the enhancement of the transcriptional level of ATP-binding cassette (ABC) transporting and two-component systems in *P. fluorescens*, indicating the potential participation of these membrane proteins during PAEs uptake (Wang et al. 2019b). A more recent study endorsed that DMP (0–40 mg/L) exposure could remove lipopolysaccharides (LPS, the major component on the cell outer membrane of *P. fluorescens*) and increase the ratio of unsaturated fatty acid/saturated fatty acid. The authors also showed that DMP could destroy the structural integrity of the lipid bilayer formed by phosphatidyl ethanolamine (PE) and phosphatidyl glycerol (PG) (Chen et al. 2022).

PAEs severely deconstruct the cell wall and membrane of Gram-negative bacteria. Similarly, their impacts on Gram-positive bacteria were also observed, although the effects were not as bad. The deconstructive effects of dimethyl phthalate (DMP, 0–80 mg/L) on the cell wall, cell membrane, and membrane proteins were identified on a typical model Gram-positive bacteria *Staphylococcus aureus* (Zhu et al. 2021). The SEM observations conducted in this study revealed a wrinkled and significantly distorted cell morphology of *S. aureus* when exposed to DMP. The bacterial cell structure displayed fractures and noticeable perforations on the cell envelope, resulting in the leakage of cellular contents at a DMP concentration of 80 mg/L. The growth of Gram-positive bacteria *Arthrobacter* QD15-4 which utilizes PAEs as the sole carbon source was not impacted by DMP even at high concentrations (400 mg/L). This indicates an alternative PAEs transport mechanism in the PAE-degrading bacteria that does not compromise the cell morphology. The authors have employed RNA-Seq and RT-qPCR experiments to show that another key change aroused by DMP exposure was the enhanced transcriptional level of genes involved in energy metabolism and ATP-binding cassette (ABC) transporters (Wang et al. 2019a).

Out of the more than 80 identified PAEs-degrading bacteria, the majority are Gram-positive bacteria. Substrate tests have revealed differences in the degradation abilities (an example shown in Table 1). Gram-negative bacteria tend to degrade low molecular weight (LMW) PAEs (with the main side chain being C1-C4), with a limited number of degradable substrate species (Hong et al. 2016; Whangsuk et al. 2015). In contrast, most Gram-positive bacteria have a broad substrate spectrum, capable of degrading a wide range of LMW and high molecular weight (HMW) PAEs (with the main side chain being C5 or more) (Huang et al. 2019; Kurane et al. 1984). Based on current research on the degradation of PAEs, Gram-positive bacteria appear to be more predominant in the degradation of these compounds. One factor that may contribute to this difference is the presence of an outer membrane in the Gram-negative bacteria. Although the outer membrane of Gram-negative bacteria seems to provide an additional selective permeability barrier against PAEs permeation (Sun et al. 2022), its delicate structure appears to be more vulnerable under PAEs exposure. The PAE-degrading Gram-positive bacteria seem to be more robust than the Gram-negative against long-side chain PAE exposure (Table 1).

**Table 1:** Degradation capacity of isolated bacteria

Strain	Sources	Gram	Main substrate	References
*Rhodococcus* sp. 2G	Active sludge	Pos	DEHP	Zhao et al. 2018
*Bacillus subtilis* BJQ0005	Baijiu starter	Pos	DiBP, DBP	Xu et al. 2021
*Artheobacter* sp. ZJUTW	River sludge	Pos	DBP	Liu et al. 2020
*Gordonia* sp. GZ-YC7	Landfill soil	Pos	DEHP	Hu et al. 2022
*Gordonia* sp. Lff	River sludge	Pos	DEHP	Huang et al. 2019
*Pseudarthrobacter defluvii* E5	Soil	Pos	DBP	Chen et al. 2021
*Rhodococcus jostii* RHA1	Soil	Pos	MMP, MBP, MHP, MEHP	Hara et al. 2010
*Gordonia* sp*.* YC-JH1	Soil	Pos	DOP	Fan et al. 2018
*Gordonia alkanivorans* YC-RL2	Soil	Pos	DEHP, DCHP, DMP, DEP, DBP	Nahurira et al. 2017
*Mycobacterium* sp*.* YC-RL4	Soil	Pos	DEHP, DCHP, DMP, DEP, DBP	Ren et al. 2016
*Pseudomonas fluoresences* FS1	Active sludge	Neg	DMP, DEP, DBP, DIBP, DOP	Zeng et al. 2004
*Providencia* sp. 2D	Compost	Neg	DBP	Zhao et al. 2016
*Sphingomonas yanoikuyae* DOS01	Ocean sediment	Neg	DMP	Gu et al. 2009
*Paracoccus kondratievae* BJQ001	Baijiu starter	Neg	DBP	Xu et al. 2020

As described above, some membrane proteins like ABC transporters are upregulated in the PAE-degrading bacterial strains. This phenomenon suggests that membrane proteins are probably participating in the bacterial uptake of PAEs. However, ABC transporters are cytomembrane proteins, and the potential involvement of membrane proteins on the outer membrane of Gram-negative bacteria should be considered separately. Although the direct transport of PAEs by outer membrane protein has not been reported, there is considerable research on the outer membrane transporters of hydrophilic PAEs analogs, such as long-chain fatty acid transport proteins (FadLs), TonB-dependent transporters (TBDTs), and outer membrane protein W family (OmpW) (Fig. 1). These channels could be involved in the transport of PAEs just like other hydrophobic matters. The participation of these membrane proteins in the transport of PAEs needs to be validated by comparing the PAE transport and degradation efficiency between the wild-type strains and the mutant strains with the gene knockout of these membrane proteins.

With a few exceptions, almost all outer membrane proteins form pore-like structures composed of 8–24 even-numbered β-strands. The number of β-strands determines their effective pore size, thereby matching the size (and shape) of solutes that can diffuse through (van den Berg et al. 2004). It is worth noting that most transporters from the porin family on the outer membrane of Gram-negative are aqueous channels that cannot transport hydrophobic aromatics like PAEs (Mutanda et al. 2022). OphP (a porin protein) from *Burkholderia* sp. was reported to transport phthalic acid through the outer membrane (Chang et al. 2009).

### Outer membrane transports in Gram-negative bacteria

#### FadL family channel

FadL family channels are widely present in Gram-negative bacteria and are known to play a definitive role in the uptake of hydrophobic molecules (long-chain fatty acids and polyaromatic hydrocarbons) across the outer membrane. The prototype member of this family, FadL, was first discovered in *Escherichia coli* (*E. coli*). The FadL protein in *E. coli*, EcFadL, is primarily responsible for the uptake and transport of long-chain fatty acids (LCFA) (Tan et al. 2017). Crystal structure analysis reveals that FadL possesses a long β-barrel structure of approximately 5 nm in length, consisting of 14 antiparallel β-folded strands. The N-terminal region of the protein, comprising 42 amino acid residues, forms a small and compact domain containing three short helices, which block the interior of the β-barrel. Between the two extracellular loops L3 and L4, there exists an exposed hydrophobic loop structure in the environment. This loop structure is considered a low-affinity binding site where free hydrophobic compounds initially interact with FadL, and it is responsible for capturing hydrophobic compounds from the environment (van den Berg et al. 2004). Three FadL family proteins (TodX, CymD, F1FadL) from *P. putida* PpF1 were proven to transport monoaromatic hydrocarbons (MAHs) through the outer membrane (Somboon et al. 2020). The authors demonstrated that MAHs molecules rapidly move from the extracellular surface to a highly affinity binding pocket near the N-terminus through a hydrophobic channel. This movement causes conformational changes in the N-terminus, restricting the MAHs in the pocket and allowing them to continue moving to the lateral opening formed by the twisted structure of S2 and S3. Finally, the MAHs enter periplasmic space (Fig. 1). However, how hydrophobic compounds move from the high-affinity pocket to the lateral opening is currently unknown. Therefore, the mechanism of FadL channel protein in transporting hydrophobic compounds may involve (1) capturing substrates from the external medium through a hydrophobic groove between the L3 and L4 loops, leading to high local concentrations; (2) diffusion of substrates to the high-affinity hydrophobic binding pocket, causing conformational changes in the N-terminus and reducing the affinity of the pocket for substrates; (3) additional conformational changes, possibly involving movement and twisting in the S3 strand, creating a lateral channel for substrate entry into the periplasmic space; (4) substrate adsorption on the inner membrane and “flipping” into the cytoplasm to complete the transport of hydrophobic compounds.

#### TonB-dependent transporter

The TonB-dependent transporter (TBDT) consists of a barrel-shaped domain composed of 22 β-folded strands spanning the outer membrane and a globular plug domain folded into the interior of the barrel (Fig. 1). When compared to the FadL family proteins, the TBDTs form larger cavities, allowing them to transport substances that are too large to diffuse through the outer membrane pore. The plug domain of TBDT contains a conserved sequence known as the TonB-box at the N terminus. The plug domain binds substrates on the extracellular side of the outer membrane, and its periplasmic region, especially the TonB-box, interacts with the TonB-ExbB-ExbD complex. The term “TonB-ExbB-ExbD” means a membrane protein complex in the inner membrane of Gram-negative bacteria. In the complex, ExbB and ExbD subunits assemble to form a heterodimer (a proton channel) and employ the proton gradient to cross the cytoplasmic membrane. The energy generated from proton transport is propagated though the TonB subunit that across the periplasm and interacts with TBDT at the outer membrane. This interaction allows the utilization of proton motive force generated by the passage of protons across the inner membrane to provide energy for the active transport of substances (Celia et al. 2019). Subsequently, the substrate in the periplasmic space is transferred to the cytoplasm through ABC transporters located in the inner membrane (Liang et al. 2021; Noinaj et al. 2010). TBDTs exhibit substrate specificity and have high-affinity substrate-binding pockets on the extracellular side of the cell membrane (Bolam and van den Berg 2018). This high-affinity binding allows TBDTs to capture low-abundance molecules from the environment, enabling bacteria to scavenge scarce nutrients. Some known or predicted TBDTs include HemR, BtuB, CirA, FatA, FcuT, FecA, FhuA, FepA, FptA, IrgA, IutA, PfeA, and PupA (Grinter and Lithgow 2020). The most extensively studied substrate transport by TBDTs is the transport of Fe^3+^-siderophore complexes (Grinter and Lithgow 2020). Increasing evidence suggests that members of the TBDT protein family transport a diverse range of substrates, including free metal ions (such as Cu^2+^ and Zn^2+^), cobalamin, polysaccharides, peptides, globular proteins, vitamin B12, and hydrophobic hydrocarbons (Nilaweera et al. 2021). Since characterized TBDTs represent only a small fraction of this protein family, the substrate diversity of this family awaits further exploration. As was reported by Liang et al., 38 TBDT genes were upregulated under the induction of benzo-a-pyrene (BaP) in *Novosphingobium pentaromativorans* US6-1. They have also observed a significantly decreased cellular fluorescent intensity of BaP in US6-1 when the TBDT-11 gene was knocked out from the genome. This indicates that TBDT should facilitate the transport of BaP (Liang et al. 2021). Another TBDT (DDVT) isolated from *Sphingobium* sp. SYK-6 is capable of transporting lignin-derived aromatic compounds. Its expression was induced by the lignin derivatives 5,5’-dehydrodivanillic acid (DDVA), but the specific transport mechanism and the substrate spectra are still unknown (Fujita et al. 2019). These works have shown that TBDT participates in the transport of hydrophobic aromatic hydrocarbons, but its role in the bacterial uptake of PAEs needs further investigation.

#### OmpW family

Another common β-barrel transmembrane protein is the OmpW family, a small outer membrane porin protein family widely found in Gram-negative bacteria. Porin proteins typically do not support the transport of hydrophobic compounds, but OmpW can mediate the transmembrane transport of small hydrophobic molecules. The most notable feature of the OmpW structure is its hydrophobic interior within the β-barrel. Taking *Escherichia coli* OmpW as an example, out of the 62 inward-facing residues in the barrel wall, only 20 are hydrophilic residues, while the rest are hydrophobic residues (Fig. 1). The residues Leu56 on the S3 strand and Trp155 on the S7 strand form a “hydrophobic gate” that controls the channel closure. In contrast to most other outer membrane proteins, the interior of the barrel on the periplasmic side of the “hydrophobic gate” exhibits stronger hydrophilic characteristics (Julsing et al. 2012). X-ray crystallography was used to find that OmpW (200–230 residues) forms an 8-stranded β-barrel structure, configuring a narrow hydrophobic channel (Hong et al. 2006).

The AlkL protein from *Pseudomonas putida* GPoI shares only 24% sequence similarity with *E. coli* OmpW. Although their protein sequence is not similar, crystal and nuclear magnetic resonance (NMR) structure analysis revealed that AlkL and OmpW share the same eight-stranded β-barrel structure, and their main difference is in the extracellular loops, also known as the β-hairpin that extends into the hydrophobic core of the crystal and the long flexible loop in solution (Schubeis et al. 2020). While the transport function of AlkL is relatively well studied, its transport mechanism is still a subject of debate. By comparing AlkL with other β-barrel transporter proteins and based on the X-ray structure of OmpW, a hypothesis has been proposed suggesting a lateral opening mechanism similar to the FadL family. However, the hypothesized lateral opening has a radius of 0.13 nm, which is incompatible with the size of known AlkL substrates such as alkanes or monocyclic molecules. Instead, Schubeis et al. utilized solid-state NMR to determine the atomic-level structure and employed molecular dynamics simulations to capture the substrate translocation process through the lateral opening. Their findings revealed a dynamic lateral extrusion mechanism involving continuous rearrangement of the extended β-barrel and transient opening to release the substrate into the lipid bilayer. This mechanism provides insights into how substrates are transported through the lateral opening in AlkL (Schubeis et al. 2020).

In summary, AlkL, OmpW, and FadL share a similar mechanism for lateral substrate transfer, allowing small hydrophobic molecules to enter the outer membrane through openings in the barrel wall. This lateral extrusion mechanism is a shared feature among these proteins, facilitating the transport of hydrophobic substrates (PAEs for example) across the outer membrane. It is important to point out that the evidence that directly shows the participation of outer membrane protein in the uptake of PAEs is still missing.

## Cytoplasm membrane transport of PAEs and analogues

For both Gram-positive and Gram-negative bacteria, cytomembrane is the last physical barrier for external compounds to enter the organism. Cytoplasm membrane is also known as cell membrane, plasma membrane, and inner membrane. It is a soft, fragile, and flexible membrane that is constituted by phospholipids (20 to 30%) and proteins (50 to 70%) that include integral membrane proteins and peripheral proteins. The adsorption of PAEs by bacteria is associated with the interactions among PAEs, phospholipids, and membrane proteins.

### PAE adsorption damages bacterial cell membrane

**Fig. 2:**
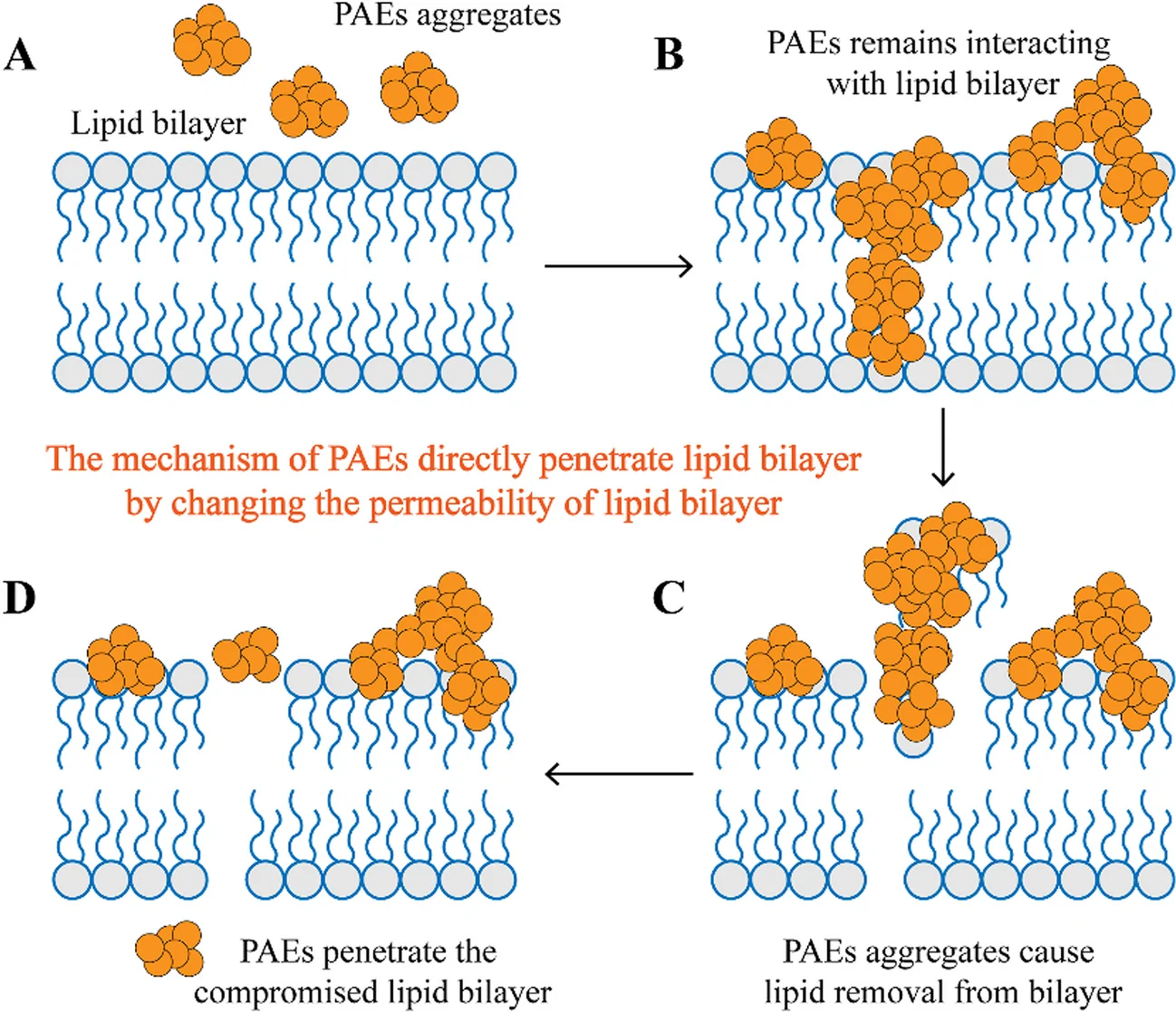
The proposed mechanism for the permeation of PAEs through lipid bilayer (Bailey-Hytholt et al. 2020). The symbol of gray circles with blue edges and tails represents phospholipids. The symbol of combined brown circles represents PAEs aggregates. **A** The state that PAEs aggregates have not bound to the lipid bilayer. **B** The state that PAE aggregates binds to the lipid bilayer. **C** The state that PAEs aggregate causes lipid removal from the bilayer. **D** The state that PAEs penetrate the lipid bilayer by migrating through the compromised structure

DMP was observed to cause serious damage to the cell membrane of *Escherichia coli* K12 and the damage was augmented with higher DMP concentrations (80 mg/L) (Wang et al. 2019c). Not only did the cell surface of *E. coli* K12 become rough and rugged, but nucleic acid leakage was also observed after DMP treatment, indicating a corruption of both the cell wall and cell membrane. As hydrophobic molecules, some PAEs could permeate cell membranes spontaneously, such as DMP, DBP, and DEHP (Bao et al. 2022). One possible mechanism is that the interaction between PAEs and the lipid bilayer has induced lipid removal. As is depicted in Fig. 2, PAEs (200 μM) first form aggregates and interact with the lipid bilayer (Bailey-Hytholt et al. 2020). At this stage, the membrane becomes thicker and more rigid. Afterward, PAEs would remove the lipid molecules bound to them and increase the permeability of the membrane bilayer. This lipid removal compromises the integrity of the membrane and could threaten the survival of cells. Finally, more PAEs traverse the damaged bilayer to penetrate the membrane, thereby posing subsequent impacts (Bailey-Hytholt et al. 2020). Differences in the interaction between PAEs with mono- and di-acyl chains and lipid bilayers were also reported (Bailey-Hytholt et al. 2020). DEHP (100 and 200 μM) could interact with the l-α-phosphatidylcholine (egg PC) bilayer to remove some egg PC molecule from the vesicle and embed itself inside to increase the hydrodynamic diameter and polydispersity of egg PC vesicles, which means that the interaction with DEHP increases the size of the egg PC vesicles. On the other hand, although its metabolite mono(2-ethylhexyl) phthalate (MEHP, 100 and 200 μM) could also induce lipid removal, it does not impact the size of the egg PC vesicles (Bailey-Hytholt et al. 2020).

It was reported that 100 μg/mL DBP, BBP, mono-n-butyl phthalate (MBP), and mono-benzyl phthalate (MBzP) could interact with the cytomembrane to embed into the bilayer and increase its permeability. This conformational change of cytomembrane has severely damaged the cell morphology and caused cell death (Sicińska 2019). Similarly, DEHP was known to migrate into the lipid bilayer and induce conformational change to the cytomembrane. Bider et al. reported that DEHP (1–9 mol %) could incorporate itself into the model POPC (1-palmitoyl-2-oleoyl-glycero-3-phosphocholine, a main component of cytomembrane) lipid membrane at very low concentration (1 mol %). The insertion of DEHP in the lipid bilayer has significantly increased the membrane width, the area per lipid, the deuterium order parameter (shows the order of the lipid membrane, the higher the deuterium order parameter is, the less fluid the lipid membranes are), and the membrane thickness. It also decreased the membrane flexibility (Bider et al. 2020). The observation made by Bao et al. that cytomembrane preferentially permits PAEs to enter the cell also supports the lipid removal mechanism (Bao et al. 2022). PAEs spontaneously enter the membrane with a preference for the headgroup-acyl chain interface. The longer the side acyl chain, the deeper the insertion was (DEHP > DBP > DMP, 9 mol%). Their explanation for this observation is the hydrophobicity of PAEs. PAEs with longer side acyl chains possess higher hydrophobicity and could interact strongly with the lipid molecules in the bilayer, this hypothesis correlates with the previous discussion on the crucial roles of hydrophobicity in the adsorption of PAEs by bacterial cell walls.

### Membrane proteins facilitated PAE transmembrane assimilation

Direct PAE permeation through cytomembrane seems to be too malignant to be the predominant strategy for the PAE-degrading strains to take, their high degradation efficiency ought to be with the assistance of other modules such as membrane proteins (Lu et al. 2008; Żyszka-Haberecht et al. 2019). As is shown in Fig. 1 and Table 2, inner membrane proteins that possibly participate in the PAEs transport are ATP-binding cassette (ABC) transporters and major facilitator superfamily (MFS) transporters (Hong et al. 2006; Prajapati et al. 2021; Rigel and Silhavy 2012; Zampolli et al. 2019).

**Table 2:** Some transporters for PAEs and the structural analogues of PAEs

Aromatic Compound	Transporter (system)	Protein family	Strains	References
PAEs, PA	PatDABC	ABC	*Rhodococcus jostii* RHA1	Hara et al. 2010
PA	OphFGH	ABC	*Burkholderia* spp.	Chang et al. 2009
Benzoic acid, 4-HBA	HbaEFGHI	ABC	*Rhodopseudomonas palustris*	Giuliani et al. 2011
PA	OphD	MFS	*Burkholderia cepacia* ATCC 17616	Chang et al 1999
4-Methylphthalate, PA	MopB	MFS	*Burkholderia cepacian* Pc701	Saint et al 1996
TPA	MucK	MFS	*Acinetobacter baylyi* ADP1	Pardo et al. 2020
Ferulic acid, PCA	VanK	MFS	*P. putida* KT2440	D’Arrigo et al. 1999
Vanillic acid, PCA	VanK	MFS	*P. putida* KT2440 *Acinetobacter baylyi* ADP1	D’Argenio et al. 1999; Wada et al. 2021

*PAEs*, phthalate esters; *PA*, phthalic acid; *4-HBA*, 4-hydroxybutyl acrylate; *TPA*, triphenylamine; *PCA*, protocatechuic acid

#### ABC transporter

So far, the only transport system reported in the literature that was identified to transport PAEs into the cytoplasm was the PatDABC ABC transport system of *Rhodococcus jostii* RHA1. However, it was found that this transport system only transported monoalkyl phthalates (MAPs, 1 mM) and phthalic acid (PA), and there was no evidence of its ability to transport dialkyl phthalates (DAPs). The ABC transporters that could transport dialkyl phthalates still await revealing. These findings suggest that there may be other transport systems involved in the uptake of DAPs in bacteria, and further research is required to identify these transport systems and understand their mechanisms of action (Hara et al. 2010). According to Hara et al., the function of *patDABC* genes was predicted through genomic analysis. Through sequence comparison and analysis of the position of *patD* within the putative pat operon, PatD was predicted to be an extracytoplasmic substrate-binding component of an ABC transporter, which belongs to the solute-binding protein (SBP) family. This analysis suggests that PatD plays a critical role in the transport of PAEs in *Rhodococcus jostii* RHA1 by binding to specific substrates and facilitating their transport across the cell membrane (Hara et al. 2007). In our previously published work, a transcription serge of ABC transporter was also observed in a Gram-positive *Gordonia* sp. GZ-YC7 that can tolerate and degrade high molecular weight PAE (DEHP) of high concentration (2 g/L) (Hu et al. 2022).

ABC transporter superfamily is the largest known primary active transport system that utilizes ATP hydrolysis to provide energy for substance transport (Hou et al. 2022). This transport system typically consists of two nucleotide-binding domains (NBDs), two transmembrane domains (TMDs), and specific solute-binding proteins (SBPs), which together form a transmembrane protein complex (Liu 2019). The NBD sequences are highly conserved, while the TMD sequences are relatively more variable, which is consistent with their respective functions. The NBD serves as an ATP-binding site that drives active transport through ATP hydrolysis (Fig. 1). The TMD recognizes specific substrates and allows their entry into the transporter protein by undergoing conformational changes (Fig. 2) (Paolini et al. 2015). In bacteria, ABC transporters are primarily responsible for the uptake of various substrates during ATP binding and hydrolysis (Facey and Kuhn 2010) (Qin et al. 2015). Another indirect evidence of the participation of ABC transporters in bacterial PAE uptake is the enhanced transcriptional level of *potACD*, *gluBC*, *oppAB*, *phdAB*, *aceAF*, and *gltA*, the genes involved in energy regulation metabolism, and ABC transporters.

#### MFS system

The major facilitator superfamily (MFS) is the largest known group of secondary transporters, which typically function as monomeric proteins, but can also form oligomers that act synergistically (Alguel et al. 2016; Li et al. 2017; Veenhoff et al. 2001). Unlike the energy supply mode of the ABC superfamily, MFS uses energy coupling with the proton motive force (PMF), also known as the electrochemical proton gradient, to accomplish its transport function (Marger and Saier 1993; Wang et al. 2020). The typical MFS has 12 α-helix transmembrane segments (TMSs) units that form two domains around the central substrate binding site to create the 3D structure of MFS (Yan 2015) (Fig. 1). During transport across the membrane, the MFS transporters undergo several conformational changes, and the substrate binding site is exposed to only one side of the membrane at a time. This mode of operation is known as the “alternating-access model” (Jardetzky 1966). Currently, the structure and transport mechanism of MFS transporters has been analyzed in more detail (Drew and Boudker 2016; Drew et al. 2021; Quistgaard et al. 2016). We have also observed a significantly increased transcriptional level of MFS transporter in *Arthrobacter* sp. ZJUTW when it was cultivated in a minimal medium with DBP as the carbon source, its log2FoldChange was 8.24 (Liu et al. 2020).

Other potential PAE transporters are membrane proteins responsible for other hydrophobic molecules such as TPA transporter MucK and SMR transporters. MucK participates in the transport of TPA, and it was identified in *Acinetobacter baylyi* ADP1 (Pardo et al. 2020). It is worth noting that some ABC transporters and MFS transporters have been shown to transport multiple substrates, possibly due to similarities in physical properties and structure between the substrates. ABC transporters appear to favor the transport of substrates with strong hydrophobicity (Henkel et al. 2022), while MFS transporters are more efficient at transporting substrates with strong hydrophilicity (such as TPA, which is insoluble in water). This difference in substrate preference may be due to the structural characteristics of the two transport systems.

## Conclusion

This comprehensive review discusses the assimilation process of PAEs in Gram-negative and Gram-positive bacteria. It covers the role of membrane proteins and spontaneous permeation through cell walls and membranes. The hydrophobic nature of PAEs highlights the importance of cell surface hydrophobicity for bacterial adsorption. Components like LPS, lipids, membrane proteins, and peptidoglycan contribute synergistically to PAE surface binding. PAE exposure adversely affects the cell wall and membrane, particularly in Gram-negative bacteria, despite their PAE degradation ability. While direct evidence is lacking, proteins like FadL, TBDT, and OmpW are implicated in transporting PAE analogs. Given the destructive effects on cell wall components, direct permeation through the outer membrane may not be the primary uptake mechanism for PAEs in degrading Gram-negative bacteria. Outer membrane proteins likely play a role in PAEs transport.

Further exploration of the involvement of these proteins in the transport of PAEs in Gram-negative strains holds great promise for future research. Once PAEs cross the cell wall, they encounter the final barrier, the cytoplasmic membrane. As discussed earlier, PAEs can extract lipid molecules from the bilayer and disrupt the membrane’s integrity. In this context, the participation of membrane proteins, specifically ABC transporters and MFS proteins, becomes particularly crucial. ABC transporters have been identified as facilitators of PAEs assimilation, with numerous studies, including our previous research, consistently reporting an upregulation of ABC transporters in response to PAE exposure. On the other hand, the role of MFS proteins in PAE transmembrane transport is still in its early stages, with indirect evidence such as increased transcription being observed.

In summary, the study of PAE bacterial assimilation remains incomplete, but the process seems to be a comprehensive process in which protein-facilitated transport is prior to direct penetration. Unraveling the specific transport mechanisms employed by membrane proteins like FadL, TBDT, OmpW, ABC transporters and MFS proteins in PAE uptake holds significant scientific importance. It would not only expand our understanding of PAE bioremediation but also contribute to the advancement of bioremediation techniques for PAE-contaminated environments. Furthermore, this knowledge could facilitate the practical application of bioremediation bacteria in PAE remediation, enabling more effective and sustainable strategies for addressing PAE pollution. 

## Data Availability

This paper is a mini-review that does not contain any experimental data.
